# Shear Speed-Regulated Properties of Long-Acting Docetaxel Control Release Poly (Lactic-*Co*-Glycolic Acid) Microspheres

**DOI:** 10.3389/fphar.2020.01286

**Published:** 2020-08-20

**Authors:** Yuhao Zheng, Fan Sheng, Zihang Wang, Guang Yang, Chenguang Li, He Wang, Zhiming Song

**Affiliations:** ^1^Department of Sports Medicine, First Hospital of Jilin University, Changchun, China; ^2^Klebs Research Center, Department of Dermatology, Yanbian University Hospital, Yanji, China; ^3^Department of Traumatology, First Hospital of Jilin University, Changchun, China; ^4^Department of Colorectal and Anal Surgery, First Hospital of Jilin University, Changchun, China; ^5^Department of Anesthesia, Yanbian University Hospital, Yanji, China

**Keywords:** shear rate, poly (lactic-co-glycolic acid) microspheres, particle size, controlled release, docetaxel

## Abstract

Advanced drug carriers for the controlled release of chemotherapeutics in the treatment of malignant tumors have drawn significant notice in recent years. In the current study, microspheres (MPs) loaded with docetaxel (DTX) were prepared using polylactic-*co*-glycolic acid copolymer (PLGA). The double emulsion solvent evaporation method is simple to perform, and results in high encapsulation efficiency. Electron micrographs of the MPs showed that controlling the shear rate can effectively control the size of the MPs. At present, most DTX sustained-release carriers cannot maintain stable and long-term local drug release. The 1.68 μm DTX-loaded microspheres (MP/DTX) with elastase was completely degraded in 14 d. This controlled degradation period is similar to a course of treatment for most cancers. The drug release profile of all kinds of MP/DTX demonstrated an initial rapid release, then slower and stable release to the end. The current study demonstrates that it is possible to create drug-loaded MPs with specific degradation times and drug release curves, which may be useful in achieving optimal treatment times and drug release rates for different diseases, and different drug delivery routes. The initial burst release reaches the effective concentration of the drug at the beginning of release, and then the drug concentration is maintained by stable release to reduce the number of injections and improve patient compliance.

## Introduction

Advanced drug carriers for the controlled release of chemotherapeutics in the treatment of malignant tumors have attracted significant attention in recent years (Wang et al., 2019). The encapsulation matrices for chemotherapy drug delivery mainly include microspheres, hydrogels (Gao et al., 2019), electrospun fibers (Ding et al., 2019c; Feng et al., 2019), nanoparticles (Ding et al., 2019b; Sun et al., 2019), nanogels (Ding et al., 2019a), and composite biomaterials (Zheng et al., 2017). Drug release systems involving microspheres (MPs) are widely used in the treatment of various diseases (García-González et al., 2015). MPs can be used as drug carriers in the nasal cavity and orally, and through injection, local administration and other modes of administration, and they are expected to be widely used in clinical practice in the future (He et al., 2013; Molavi et al., 2020).

The carrier material in MPs has a direct relationship with their properties. An MP carrier material must meet the following requirements: good biocompatibility, non-toxicity, ideal drug dissolution and diffusion abilities, excellent drug compatibility, suitable processing performance, simple preparation process, and low production cost (Prajapati et al., 2015). Many carrier materials can be used to prepare polymer MPs (Floyd et al., 2015; Annamalai et al., 2018). Among these, poly (lactic-co-glycolic acid) (PLGA) has attracted significant attention as a biodegradable synthetic polymer (Han et al., 2009; Kapoor et al., 2015). Using PLGA to prepare MPs has advantages compared with traditional treatment methods, and such MPs can slow the release rate of drugs and prolong the activity of drugs to improve their bioavailability (Minardi et al., 2020). PLGA is currently widely used as a drug carrier and bio-scaffold (Wu et al., 2010; Li et al., 2019), and it is also a biodegradable polymer authorized by the US Food and Drug Administration (FDA) for injection (Makadia and Siegel, 2011). The precursors of most drugs are fat-soluble. Researchers have chemically modified drugs to increase their water solubility, which affects their efficacy. PLGA MPs can contain many small molecule drugs, such as 5-fluorouracil, cisplatin, dexamethasone, docetaxel (DTX), doxorubicin, and paclitaxel (Floyd et al., 2015; Zheng et al., 2017). They have a higher drug loading rate and encapsulation rate for fat-soluble drugs. With the gradual degradation of the PLGA matrix, PLGA MPs can release a drug more stably and can achieve long-term continuous drug delivery. DTX is widely used for chemotherapy of a variety of tumors, and has a good curative effect. In the current study, DTX was used as a representative drug to study the controlled release of drugs from MPs.

DTX, also known as Taxol or Taxotere, is a new antitumor drug. The structure and effects of DTX are similar to those of paclitaxel, and it is a mitotic inhibitor. It promotes tubulin aggregation and inhibits tubulin depolymerization to prevent cells from undergoing normal mitosis, and also promotes apoptosis. In clinical practice, DTX, alone or in combination with other chemotherapeutic drugs, has begun to be used to treat breast cancer, lung cancer and other tumors, and it has a good therapeutic effect with few side effects (Ashrafizadeh et al., 2019). DTX is a fat-soluble drug with poor water solubility. DTX solution for injection contains a large amount of Tween, which is likely to cause severe allergic reactions in clinical applications (Tan et al., 2012). In addition, because of significant toxic and side effects, it is indispensable to exploit a novel DTX drug delivery system (Gong et al., 2020). Drug delivery systems for DTX mainly exploit liposomes, albumin and other nanoparticles as carriers (Gong et al., 2020). Although these nanoparticle carrier systems have unique targeting properties, improve drug stability, and reduce toxic and side effects, their release time is relatively short, whereby the shortest is 1–2 days, and the longest is approximately 2 weeks (Musumeci et al., 2006; Hwang et al., 2008). Therefore, it is necessary to develop a drug carrier with a longer release time.

The current methods for preparing MPs include solvent extraction, phase separation, solvent evaporation, spray drying, and supercritical fluid technology. In the current study, the solvent evaporation method was applied. The basic principle of this method is to prepare an emulsion of two immiscible oil-water phases through mechanical stirring or ultrasonication. The organic solvent in the interior phase is volatilized and the droplets are solidified into MPs. The emulsion solvent evaporation method is currently the most common technique for preparing MPs. The process for this technique can be separated into the following procedures: First, the polymer is dissolved in a volatile and water-soluble organic solvent (such as methylene chloride), and the drug is dissolved or dispersed in the polymer material solution. Second, the mixed solution or suspension is emulsified in the continuous phase to formulate uniformly dispersed emulsion droplets. Finally, the organic solvent is volatilized under constant stirring, and the dispersed emulsion droplets are solidified into spheres, which are then precipitated through centrifugation to obtain MPs.

The process can be a two-phase emulsion system or a multi-phase emulsion system based on the number of immiscible phases during emulsion formation (Perez et al., 2000). A two-phase emulsion system means that the emulsion is emulsified and dispersed from two immiscible phases. The most common method is the oil-in-water (O/W) method. A multi-phase emulsion system is an emulsion made by emulsifying three or more phases, such as the water-in-oil-in-water (W/O/W) method. MPs are mostly prepared through the O/W method for fat-soluble drugs or drugs with low solubility in water. The W/O/W method is used to encapsulate hydrophilic drugs, especially protein drugs (Utzinger et al., 2017). The solvent evaporation method is simple to perform, easy to control, and has outstanding reproducibility. The mild conditions in the preparation process of this method are difficult to destroy the stability and biological activity of the drug, and the drug utilization rate is high. The solubility of a drug in an organic solvent will affect the distribution of the drug in the MPs and the drug load. Because of the high solubility of DTX in the organic phase, drug-loaded PLGA MPs were directly compounded using the O/W method in the current study.

Particle size and distribution are the most critical MP variables. The main factors that affect the MP particle size are the stirring speed and the shape of the stirrer. The stirring speed has the largest effect on the average particle size of MPs. Generally, the particle size of drug-loaded MPs decreases with an increase in the stirring rate (Kim et al., 2002; Bagheri-Khoulenjani et al., 2013).

There are also many unknown factors concerning PLGA MPs, such as whether the addition of fat-soluble drugs will affect the particle size of the MPs; how to effectively control the degradation time and drug release rate of drug-loaded MPs to adapt to the course of different diseases and different routes of administration. Through several preliminary experiments, it was difficult to form microspheres when the shear rate was less than 1,000 r/min. However, the particle size of the microspheres formed by shearing too fast was less than 1 μm, which leaded to an increase in the surface area of the microspheres, and the degradation and release rate were excessively fast. At the same time, the drug diffusion distance was too short, which also caused the drug release rate to be overquick. Therefore, we chose shear speed of 1,000 r/min to 3,500 r/min for systematic research, to prepare PLGA MPs with various drug release rates.

## Materials and Methods

### Materials

PLGA (intrinsic viscosity (*η*) = 0.6 dL g^−1^, LA: GA = 75:25, mol/mol) was purchased from Changchun SinoBiomaterials Co., Ltd. (Changchun, P. R. China). DTX was purchased from Beijing Huafeng United Technology Co., Ltd. (Beijing, P. R. China). Poly (vinyl alcohol) (PVA), sodium hydroxide (NaOH), sodium dodecyl sulfonate (SDS), and dichloromethane were obtained from Shanghai Chemical Reagent Co., Ltd. (Shanghai, P. R. China). Elastase was acquired from Aladdin Reagent Co., Ltd. (Shanghai, P. R. China).

### Synthesis of Blank MPs and MP/DTX With Different Particle Sizes

The blank MPs and DTX-loaded MPs (MP/DTX) were compounded through the O/W solvent evaporation method. The dosage of DTX was uniformly 10%. For the preparation of MP/DTX, 135.0 mg of PLGA, and 24.0 mg of Tween-80 were dissolved in 4.5 mL of dichloromethane containing 15.0 mg of DTX. After the complete dissolution of PLGA and DTX, the mixture was slowly injected into 50.0 mL of PVA aqueous solution with a mass fraction of 1.0 wt. % over 3 min. At the same time, the PVA solution was sheared at a rotating speed of 1,000 r/min using a high-speed shearing machine. After pouring the emulsion into 100.0 ml of double distilled water and stirring for 6 h, the dichloromethane was fully volatized. Next, the products were centrifuged and collected at 2,000 r/min for 3 min, and then washed using double distilled water three times. After lyophilization, the obtained white powder was MP/DTX, created using a rotating speed of 1,000 r/min.

The shear speed of the high-speed shearing machine was adjusted to prepare MP/DTX with a rotating speed of 1,500, 2,000, 2,500, 3,000, and 3,500 r/min (all other steps were consistent with those in the procedure used to create MPs at 1,000 r/min). Blank MPs were prepared using a similar procedure without DTX.

### Drug-Loading Content, Efficiency, and Solid Matter Yield of MP/DTX With Different Particle Sizes

A sodium hydroxide−sodium dodecylsulfonate (NaOH−SDS) method was used to measure the drug-loading content (DLC) and drug-loading efficiency (DLE) of the MP/DTX. Lyophilized microsphere powders (2.0 mg, three groups) were dissolved in 1.0 mL of a NaOH−SDS (5 wt.%, NaOH 0.1 mol L^−1^) solution and shaken overnight at a constant temperature of 37°C in an oscillation box (HZQ-X100, Donglian Co., Ltd., Harbin, P. R. China). After the decomposition of MP/DTX, the clear liquid supernatant was removed, and the DTX concentration in the supernatant was measured through high-performance liquid chromatography (HPLC); (Waters 1525 system with a Waters C18 column and a Waters 2489 ultraviolet/visible (UV/vis) detector, Waters, Milford, MA, USA). A DTX standard curve was established. The elution was acetonitrile−water (60:40, V/V). And the flow rate of elution was 1.0 ml min^−1^. The absorption wavelength (λabs) of DTX was set at 230 nm. The data was calculated by Breeze software. The mass of DTX in the MPs was calculated and the measurement was repeated three times. Then DLC, DLE, and solid matter yield were calculated using formula (1), (2), and (3).

(1) $$\text{DLC}(\%)=\frac{W_{\text{loaded drug}}\text{ (mg)}}{W_{\text{loaded MP}}\text{ (mg)}}\times 100\%$$

Where, *W*_loaded drug_ and *W*_loaded MP_ denote the weight of loaded drug and MP/DTX, respectively.

(2) $$\text{DLE }(\%)=\frac{W_{\text{loaded drug}}\text{ (mg)}}{W_{\text{feeding drug}}\text{ (mg)}}\times 100\%$$

Where, *W*_loaded drug_ and *W*_feeding drug_ denote the weight of the loaded drug in the MP/DTX and the feeding drug in the course of drug encapsulation, respectively.

(3) $$\text{Yield}(\%)=\frac{W_{\text{loading MP}}\text{ (mg)}}{W_{\text{feeding solid}}\text{(mg)}}\times 100\%$$

Where, *W*_feeding solid_ denotes the weight of the entire feeding solid in the course of drug encapsulation.

### Morphology and Particle Size Measurement of Blank MPs and MP/DTX With Different Particle Sizes

Blank MPs and MP/DTX with different particle diameters were pasted to different areas of a metal plate using conductive adhesive tape, and then sprayed with gold. A scanning electron microscope was used to observe the morphology of the MPs, and 100 MPs in each group were selected and the average particle diameters were measured. Then the variance of the particle diameters was calculated.

### *In Vitro* Degradation of MP/DTX With Different Particle Sizes

MP/DTX was added into 10 mL centrifuge tubes (30.0 mg for each group), and then 5.0 mL of PBS without or with 2.0 mg ml^−1^ of elastase was laxly put in the tubes. Then the tubes were placed into an oscillation chamber at 37°C. The vibration rate was set to 70 rpm. The tubes were centrifuged at 1,000 r min^−1^ every 7 d, and then the supernatant was aspirated. After lyophilization, the total mass of the centrifuge tubes was measured. Finally, the new buffer was replaced, and then shaking at 37°C continued. Three parallel control tubes were set up per group. When the MP powder of each particle size had completely degraded, a line chart of the remaining mass and days was constructed.

### *In Vitro* Release Profiles of MP/DTX With Different Particle Sizes

The dynamic direct release method was used to obtain a drug release curve of MP/DTX with different particle sizes. First, 2.0 mg of MP/DTX with different particle diameters was placed into centrifuge tubes. Then 2.0 ml of PBS without or with elastase (2.0 mg ml^−1^) was put in the tubes. Three parallel controls per group were established. After the tubes were sealed, they were placed into an oscillation chamber at 37°C. The vibration rate was set to 70 rpm. The tubes were centrifuged at 1,000 r/min for 5 min to separate MP/DTX and the release solution every other day. The release solution was filtered through a micro-porous filter membrane, and the released drug concentration was measured through HPLC. When the MP/DTX had completely degraded, fresh buffer was added to the tubes and the previous steps were repeated. The total time of degradation and drug release was recorded for each group, and drug release curves were obtained based on the time and cumulative release.

### Statistical Analyses

All the statistical data are shown as mean ± standard deviation. Differences were analyzed using the paired Student’s *t*-test. Differences between experimental groups were assessed by one-way analysis of variance with statistical software SPSS 17.0 (SPSS Inc.,Chicago, IL). *P* < 0.05 was considered statistically significant, *P* < 0.01 was considered highly significant, and *P* > 0.05 was considered no significant.

## Results and Discussion

### Morphology and Particle Size Analysis of Blank MPs and MP/DTX With Different Particle Sizes

**Figure 1:**
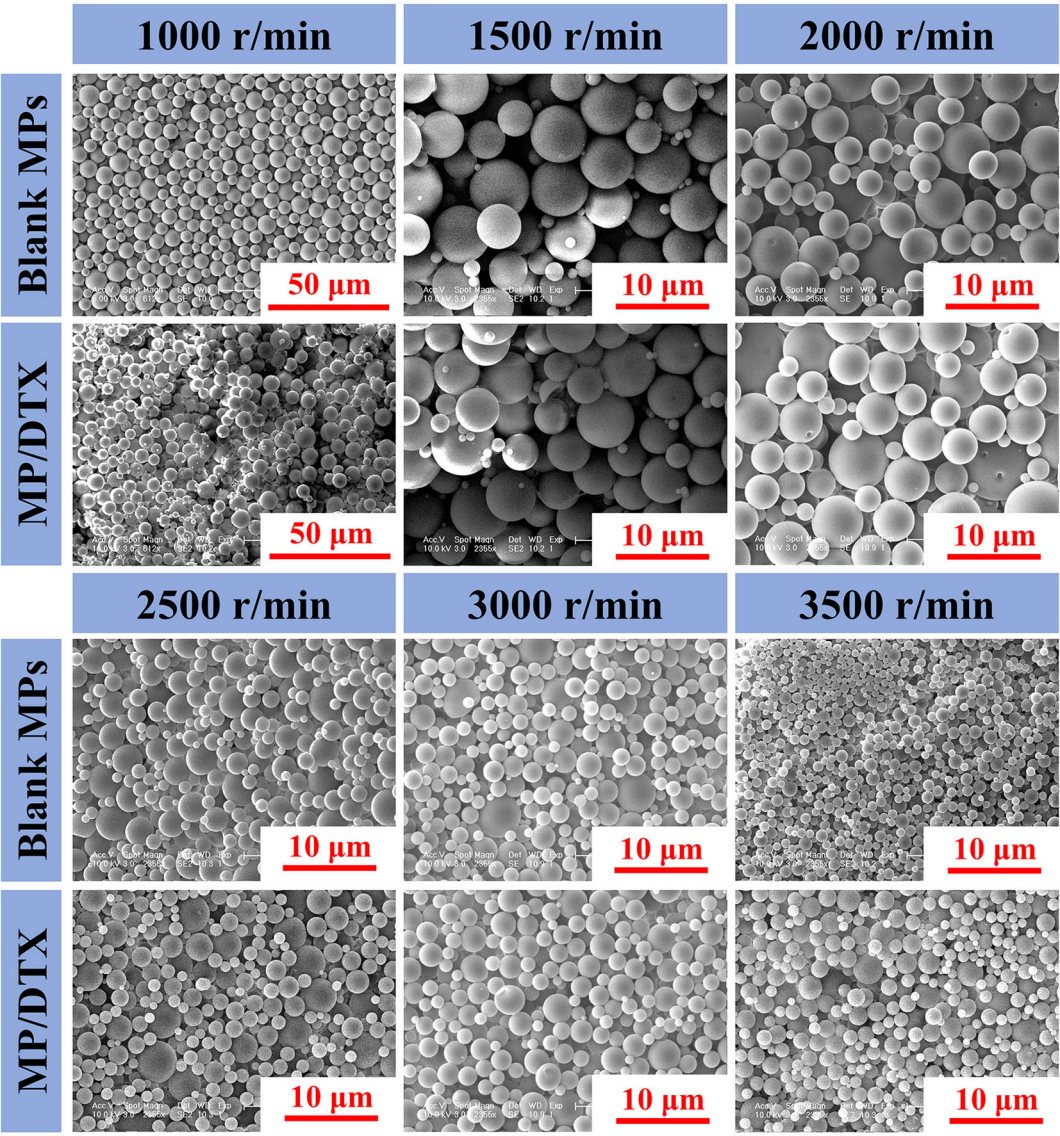
Morphology and scale of blank microspheres (MPs) and MP/docetaxel (DTX) created using different shear speeds.

As shown in **Figure 1**, the morphology of blank MPs and MP/DTX with different particle sizes was observed through scanning electron microscopy (SEM; Philips XL30, Eindhoven, The Netherlands). All the MPs were perfectly round spheres with smooth surfaces. The average size of the MPs was analyzed by assessing 100 MPs, using the scale bar in the **Figure 1**. The mean and variance of the particle size were calculated, and the results are shown in **Table 1**. When the emulsification speed increased from 1,000 r/min to 3,500 r/min, the average particle size of the blank MPs decreased from 8.84 μm to 1.53 μm, and the average particle size of MP/DTX decreased from 8.98 μm to 1.68 μm. This indicates that the shear rate has a prominent effect on the particle size of both blank MPs and MP/DTX (*P* < 0.01). When the shear rate is increased, the degree of dispersion of the oil phase droplets in the water phase increases, causing a smaller particle size in the formation of MPs. The particle size distribution is also narrowed to some extent. The particle size and distribution of MPs created using different shear rates were very close, regardless of whether DTX was added (*P* > 0.05). These results show that without changing the dichloromethane concentration in the PLGA solution, adding fat-soluble drugs has little impact on the particle size of the MPs.

**Table 1:** Particle sizes of blank microspheres (MPs) and MP/docetaxel (DTX) created using different shear speeds.

Shear Rate(r/min)	Particle Size of blank MPs(μm)	Particle Size of MP/DTX(μm)
1,000	8.84 ± 1.81^a,b^	8.98 ± 1.53^a,c^
1,500	6.61 ± 1.86^a,b^	6.73 ± 1.28^a,c^
2,000	4.81 ± 1.33^a,b^	4.85 ± 1.20^a,c^
2,500	3.34 ± 1.01^a,b^	3.47 ± 1.41^a,c^
3,000	2.64 ± 0.67^a,b^	2.56 ± 0.74^a,c^
3,500	1.53 ± 0.32^a,b^	1.68 ± 0.36^a,c^

Data are presented as mean ± SD (n = 100, ^a^P > 0.05, ^b^P < 0.01, ^c^P < 0.01).

### Drug-Loading Content, Efficiency, and Solid Matter Yield of MP/DTX With Different Particle Sizes

As shown in **Table 2**, when the dosage of DTX was consistent (10 wt. %), the DLC decreased from 8.95% to 8.63% when the emulsification rate increased from 1,000 r/min to 3,500 r/min. This is because with the increase in the emulsification and dispersion rate, the surface area of the droplets increases, and the diffusion distance of the drugs decreases. A small amount of DTX is lost, so the DLC decreases (Berkland et al., 2003). However, because of the strong lipid solubility of DTX, the DLC and DLE of each group was very high without significant differences (*P* > 0.05). Moreover, the yield for each MP/DTX group was above 85%, which indicates that the loss of material during the volatilization solvent evaporation method is minimal. Therefore, these PLGA MPs demonstrate controlled size, morphology and high DLE, and are a promising platform to encapsulate and deliver drugs such as DTX.

**Table 2:** Particle sizes, drug-loading content, efficiency, and solid matter yield of microsphere (MP)/docetaxel (DTX).

Shear Rate(r/min)	Drug Loading (%)	Loading Efficiency (%)	Yield (%)
1,000	8.95 ± 0.26*	76.57 ± 4.19	85.55 ± 3.22
1,500	8.94 ± 0.38*	77.89 ± 3.58	87.12 ± 2.94
2,000	8.92 ± 0.31*	78.47 ± 2.75	87.97 ± 1.51
2,500	8.90 ± 0.19*	79.95 ± 2.59	89.83 ± 1.76
3,000	8.88 ± 0.15*	80.21 ± 1.93	90.33 ± 2.85
3,500	8.63 ± 0.29*	79.53 ± 2.69	92.15 ± 2.36

Data are presented as mean ± SD (n = 3, *P > 0.05).

### *In Vitro* Degradation of MP/DTX With Different Particle Sizes

**Figure 2:**
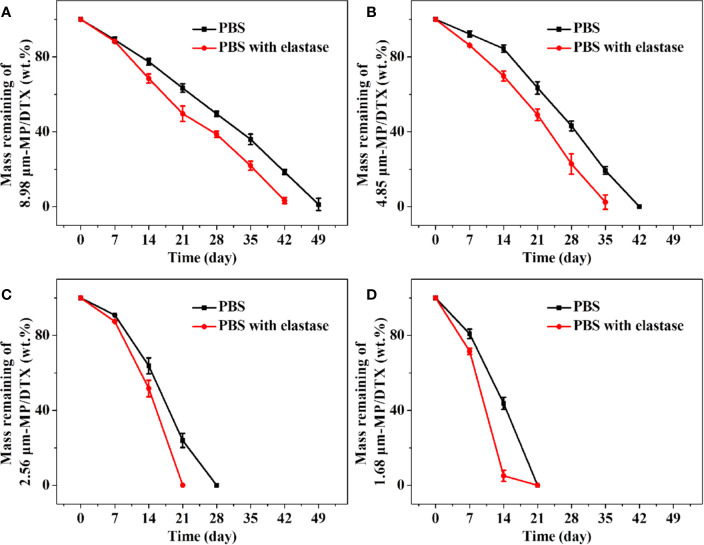
Degradation curves of remaining masses of 8.98 μm microsphere (MP)/docetaxel (DTX) **(A)**, 4.85 μm MP/DTX **(B)**, 2.56 μm MP/DTX **(C)**, and 1.68 μm MP/DTX **(D)**. Data are presented as mean ± SD (n = 3).

The following conclusions can be drawn from **Figure 2**. Firstly, elastase was added to mimic the *in vivo* circumstances, which promoted the degradation of the MPs. Secondly, the MPs with the smallest particle size obtained at high shear rates had the fastest degradation rate and the shortest degradation time. The 1.68 μm MP/DTX with elastase was completely degraded in about 14 d, while 2.56 μm MP/DTX, 4.85 μm MP/DTX and 8.98 μm MP/DTX with the same elastase were degraded in 21, 35 and 42 d. Most tumor chemotherapy takes about 14 days in one course of treatment. At this time, 1.68 μm MP/DTX can be applied. If it is necessary to prolong the chemotherapy time according to the condition, it can be treated by MP/DTX with a longer degradation time. Finally, all degradation curves were slower in the early stage and faster in the later stage. This is because the PLGA molecular chain contains a hydrophilic ester bond, which can hydrolyze in an aqueous medium, causing the polymer molecular chain to break. The hydrolytic cleavage of the ester bond is random, and the polymer chain was broken into low molecular weight chains. However, these low molecular weight chains still demonstrate a certain degree of polymerization and can be bonded to each other. Consequently, at this stage, the mass of the MPs did not change much. With the further degradation of the MPs, the short chain molecules continued to hydrolyze into acidic small molecule monomers such as lactic acid, and entered the aqueous medium through channels such as molecular chain gaps and pores. Finally, with the continuous loss of small molecule monomers, the MPs gradually dissolved and disintegrated until they disappeared completely. Therefore, the degradation of the MPs changed significantly in the later stage. (Mogi et al., 2000) In addition, polyester materials are also affected by their own degradation products. The degradation products were small molecules of lactic acid and glycolic acid. It was difficult for such molecules to diffuse to the outside of the matrix, which caused the accumulation of acid degradation products inside the MPs, forming a local slightly acidic environment. Hydrogen ions can catalyze the hydrolysis of ester bonds, which is called the “autocatalytic effect”, which also leads to the faster degradation of MPs in the later stage (Busatto et al., 2018).

### *In Vitro* Release Profiles of MP/DTX With Different Particle Sizes

The release performance of drug-loaded MPs *in vitro* is an important index in terms of evaluating MP performance and effectiveness. The advantages of the method used in the current study are its simple operation and slow shaking speed. MPs are piled up on the bottom of the container. MPs in this state mimic the condition of MPs when injected subcutaneously or intramuscularly. The main disadvantage of this method is that some samples may be lost during sampling, which will increase the relative error of the *in vitro* release measurement.

**Figure 3:**
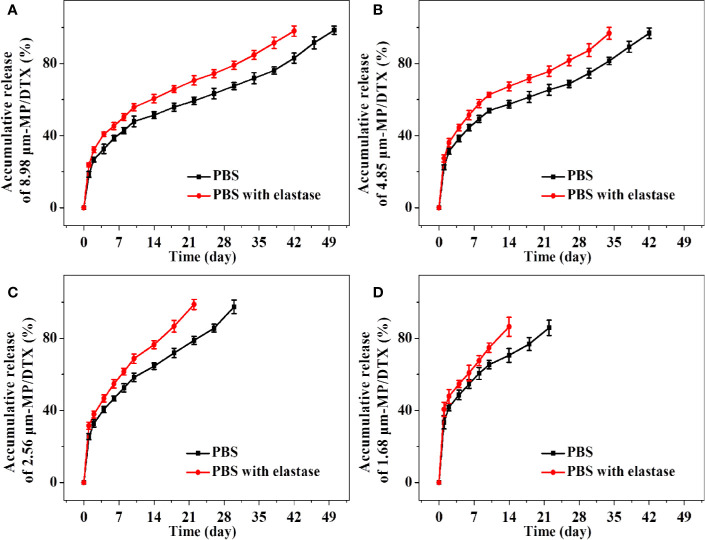
Accumulative release amount and release time of docetaxel (DTX) for 8.98 μm microsphere (MP)/DTX **(A)**, 4.85 μm MP/DTX **(B)**, 2.56 μm MP/DTX **(C)**, and 1.68 μm MP/DTX **(D)**. Data are presented as mean ± SD (n = 3).

As shown in **Figure 3**, the groups of MP/DTX with elastase had finished release 1 week earlier than the group without elastase, which is consistent with the results of the MP degradation experiments. The drug release profile of all kinds of MP/DTX demonstrated an initial rapid release, then slower release, and then slightly accelerated release. The degradation trend of 2.56 μm MPs in PBS was taken as an example. 47.86% of the total DTX was desorbed quickly in the first ten days. DTX was released in a smooth and slow trend from the 10th day to the 38th day. During this period, 1.01% of the total DTX was released every day. From the 38th day to the 50th day, DTX was released rapidly with an average daily release of 1.86% of the total DTX. Based on this trend, the process of drug release from the MPs can be divided into three phases: an initial burst release phase, a stable release phase, and a late accelerated release phase.

In the first stage, an initial burst release can increase the blood concentration quickly to reach the effective therapeutic concentration, which is beneficial for treatment. However, an excessive burst release may easily lead to the blood concentration approaching toxic levels, which may cause adverse reactions. This “bursting” effect can be reduced by washing the MPs multiple times during the preparation course. The surface morphology and particle size of the MPs are the main factors affecting the initial burst release (Jeyanthi et al., 1997). As shown in **Figure 3**, with the decrease in the particle size of the MP/DTX, the initial burst release increased. The cumulative DTX release of the four MP types (with elastase), from a large to small particle size, in the first 2 d was 23.70%, 27.37%, 31.45%, and 40.53%, respectively. As the particle size of the MP/DTX decreased, the surface area was larger, resulting in an increase in the contact area between MP/DTX and the degradation solution. Therefore, the smaller the MP/DTX particle size, the greater the initial burst release phenomenon. Secondly, when the hydrophilicity of the drug is low, the drug that diffused out of the MPs will adhere to the MP surface during the volatilization of the solvent (Rieger et al., 2015).

The initial burst release of the four MPs occurred in the first 2 d, and then the stable release phase began. During this process, the external liquid gradually diffused to the inside of the MPs and drove the drug to diffuse outward. At the same time, the polymer carrier was gradually degraded, which drove the diffusion of external liquids into the MPs and the diffusion of drugs outside the MPs. This process is controlled by both the diffusion process and the degradation process.

In the last stage of drug release, when the matrix degradation of the polymer reached a certain level, the MPs completely swell and disintegrated completely, so that the unreleased drugs are fully released. Although the complete disintegration of the MP/DTX accelerated the release of DTX, the external liquid had gradually diffused into the MP/DTX during the stable release phase, taking away most of the internal DTX, and the release rate did not change a lot.

## Conclusions

In the current study, PLGA MPs loaded with DTX were prepared through the solvent evaporation method. By observing electron micrographs of the MPs, it was found that controlling the shear rate can effectively control the particle size of the MPs. The obtained MPs have a complete glossy surface and a standard spherical shape. These PLGA MPs also have a high drug loading rate, high yield, and they are easy to prepare and adjust the MP particle size. The degradation and release characteristics of MPs with different particle sizes were determined through degradation and release tests. It is conducive to selecting the appropriate drug-loaded MPs, which match the degradation time with the time of therapy according to the needs of different disease. This method can carry not only DTX but also other anti-tumor drugs. Simultaneously, as an excellent drug delivery vehicle, it can continuously release the drug locally in the tumor for a long time, thereby maintaining the local concentration of the drugs. Therefore, compared to systemic medicine, this drug delivery vehicle can reduce the toxic and side effects by locally releasing the drug near the tumor. This new drug delivery system will play a more effective role in chemotherapy for human tumors.

## Data Availability Statement

All datasets generated for this study are included in the article/supplementary material.

## Author Contributions

All authors contributed to the article and approved the submitted version.

## Funding

The current study was financially funded by the National Natural Science Foundation of China (No. 81902227); the China Postdoctoral Science Foundation (No. 2018M631864); the National Postdoctoral Program for Innovative Talents of China (No. BX201701278); and the Department of Science and Technology of Jilin Province of China (Nos. 20190303154SF, 20200201478JC).

## Conflict of Interest

The authors declare that the research was conducted in the absence of any commercial or financial relationships that could be construed as a potential conflict of interest.
